# Automated grading system of retinal arterio-venous crossing patterns: A deep learning approach replicating ophthalmologist’s diagnostic process of arteriolosclerosis

**DOI:** 10.1371/journal.pdig.0000174

**Published:** 2023-01-11

**Authors:** Liangzhi Li, Manisha Verma, Bowen Wang, Yuta Nakashima, Hajime Nagahara, Ryo Kawasaki

**Affiliations:** 1 Institute for Datability Science (IDS), Osaka University, Osaka, Japan; 2 Graduate School of Medicine, Osaka University, Osaka, Japan; Yonsei University College of Medicine, KOREA, REPUBLIC OF

## Abstract

The morphological feature of retinal arterio-venous crossing patterns is a valuable source of cardiovascular risk stratification as it directly captures vascular health. Although Scheie’s classification, which was proposed in 1953, has been used to grade the severity of arteriolosclerosis as diagnostic criteria, it is not widely used in clinical settings as mastering this grading is challenging as it requires vast experience. In this paper, we propose a deep learning approach to replicate a diagnostic process of ophthalmologists while providing a checkpoint to secure explainability to understand the grading process. The proposed pipeline is three-fold to replicate a diagnostic process of ophthalmologists. First, we adopt segmentation and classification models to automatically obtain vessels in a retinal image with the corresponding artery/vein labels and find candidate arterio-venous crossing points. Second, we use a classification model to validate the true crossing point. At last, the grade of severity for the vessel crossings is classified. To better address the problem of label ambiguity and imbalanced label distribution, we propose a new model, named multi-diagnosis team network (MDTNet), in which the sub-models with different structures or different loss functions provide different decisions. MDTNet unifies these diverse theories to give the final decision with high accuracy. Our automated grading pipeline was able to validate crossing points with precision and recall of 96.3% and 96.3%, respectively. Among correctly detected crossing points, the kappa value for the agreement between the grading by a retina specialist and the estimated score was 0.85, with an accuracy of 0.92. The numerical results demonstrate that our method can achieve a good performance in both arterio-venous crossing validation and severity grading tasks following the diagnostic process of ophthalmologists. By the proposed models, we could build a pipeline reproducing ophthalmologists’ diagnostic process without requiring subjective feature extractions. The code is available (https://github.com/conscienceli/MDTNet).

## Author Summary

Assessment of arterio-venous crossing points in retinal images provides rich cues for quick screening of arteriosclerosis and even for classifying them into different severity grades. Considering the ever-increasing demand for ophthalmologic examination, computer-aided diagnosis (CAD) is extremely helpful for quick screening. However, retinal image analysis for CAD is a challenging task due to the high complexity of the vessel system and huge visual differences among retinal images. To address the aforementioned problems, we propose a whole pipeline for an automatic method for severity grading of artery hardening. Our method can find and validate possible arterio-venous crossing points, for which the severity grade is predicted. We also design a new model, MDTNet, which uses the focal loss to address the problem of data ambiguity and unbalance. Therefore, we believe that this research contributes to the advancement of research in machine learning on retinal images.

## Introduction

Retina provides a window to directly visualize vascular structure *in vivo*, and ophthalmologic examination has been regarded as an important routine for detecting not only eye diseases but also ocular manifestations of cardiovascular diseases or their accumulated risks [1]. Among these detectable retinal vascular signs, arteriolosclerosis is critical yet asymptomatic, of which diagnosis requires detailed retinal observation. It is not widely conducted in the modern medical practice as it depends on mostly subjective qualitative observations, and most importantly, it requires vast experiences.

**Fig 1:**
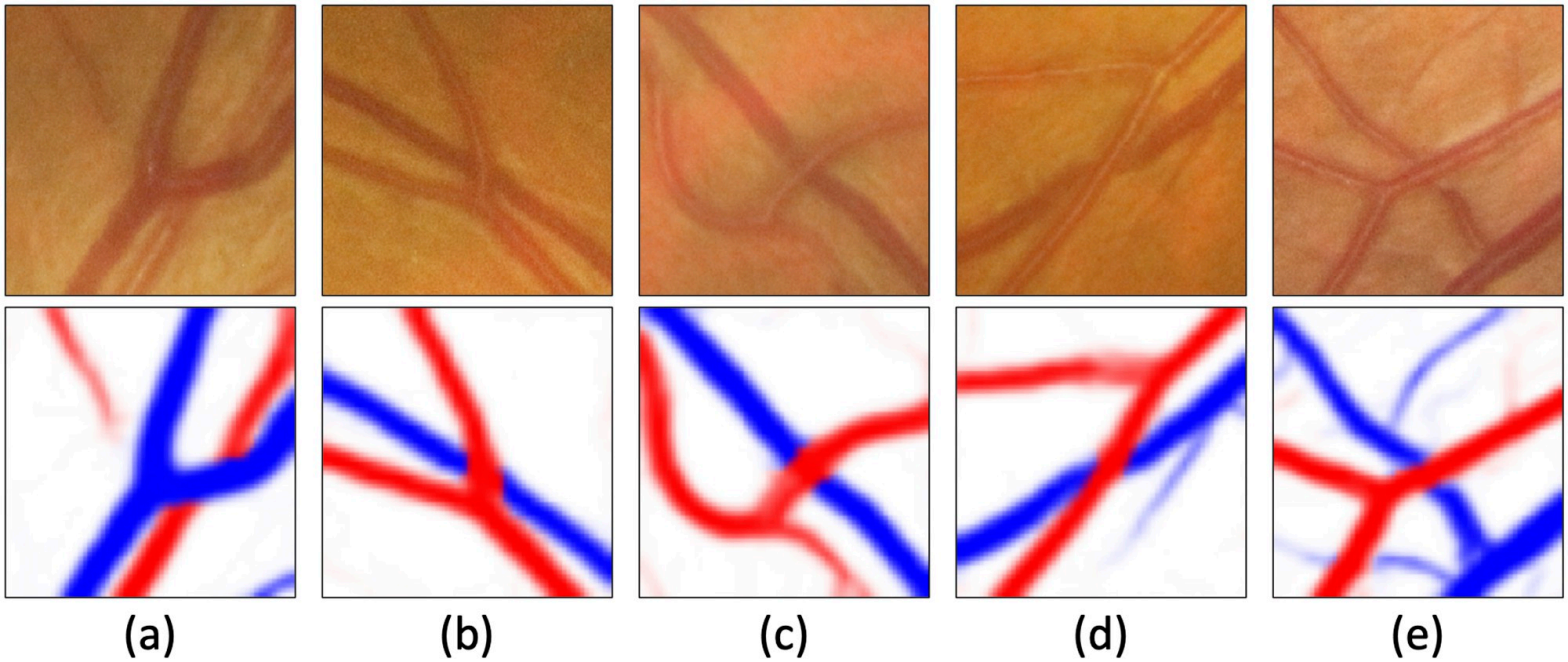
Typical examples of our prediction targets. Images in the first and second rows are raw retinal patches and automatically-generated vessel maps with manually-annotated artery/vein labels, respectively. Red represents arteries while blue represents veins. (a) is false crossing (the vein runs above the artery), while (b)–(e) are for *none*, *mild*, *moderate*, and *severe* grades, respectively. Note that even the state-of-the-art segmentation techniques cannot capture caliber narrowing, therefore, the arterioloscleroses are not very obvious in the vessel maps.

Assessment of arterio-venous crossing points in retinal images provides rich cues for screening arteriosclerosis and for evaluating accumulated cardiovascular risks. Typically, arterio-venous crossing points are classified into severity grades [2]. The assessment is based on some diagnostic criteria, for example, Scheie’s classification [3], as shown in Fig 1(b)–1(e). The grades are described as follows: (i) *none* (no anomaly observed); (ii) *mild* (slight shrink in the caliber at venular edges); (iii) *moderate* (narrowed caliber at a single venular edge); and (iv) *severe* (narrowed caliber at both venular edges).

However, human graders are subjective and usually with different levels of experience, and there has been a criticism of the low reproducibility of severity grading, which makes grading results from human graders unreliable for clinical practice, screening, and clinical trials [4]. Also, considering the ever-increasing demand for ophthalmologic examination, computer-aided diagnosis (CAD) is extremely helpful for quick screening. Yet, retinal image analysis for CAD is a challenging task due to the high complexity of the vessel system and huge visual differences among retinal images.

In fact, most researchers in this area have been focusing on preliminary tasks, such as vessel segmentation [5–7], artery/vein classification [8–10], etc. A few works address higher-level tasks [4, 11], mostly on top of vessel segmentation, such as vessel width measurement, vessel-to-vessel ratio calculation, etc. However, they usually struggle in actual diagnoses: Firstly, vessel segmentation in retinal images *per se* is a challenging task. The vessel maps in Fig 1(c)–1(e), which are produced by the state-of-the-art segmentation model [12], cannot capture such deformation. This may imply that deformation is too minor to be captured by segmentation models, although such kind of segmentation-based approach is a typical solution for automatic severity grading. Secondly, the existing methods detect arterio-venous crossing points by applying some morphological operators to vessel maps [13]. This approach may not be accurate enough to find crossing points that satisfy diagnostic requirements. For example, we can only use crossing points at which the artery is above the vein for diagnosis, and Fig 1(a) is not a diagnostic crossing point since the artery goes below the vein.

Instead of fully relying on segmentation results, we propose a multi-stage approach, in which segmentation results are used only for finding crossing point candidates, and actual prediction of the severity grade is conducted for an image patch around each crossing point after validating if the crossing point is an actual and informative one. To the best of our knowledge, this is the first work proposing a fully-automatic methodology aiming at grading arteriolosclerosis through the joint detection and analysis of retinal crossings.

Another issue in our severity grading task, which is very common in medical imaging, is the imbalanced label distribution. Most patients in our dataset have the slightest signs (*none* and *mild*) of arteriolosclerosis while only a few patients suffer from the *severe* grades of artery hardening. Also, the boundaries among different severity labels are not always obvious, making accurate diagnosis challenging.

Inspired by the concept of the multidisciplinary team [14], which strives to make a comprehensive assessment of a patient, we propose a multi-diagnosis team network (MDTNet) in this paper to address the imbalanced label distribution and label ambiguity problems at the same time. MDTNet can combine the features from multiple classification models with different structures or different loss functions. Some of the underlying models in MDTNet use the class-balanced focal loss [15] to handle hard or rare samples, of which the original version requires hyperparameter tuning, while MDTNet can utilize the advantage of the focal loss without tuning its hyperparameters.

Our main contribution is two-fold: (i) We propose a whole pipeline for an automatic method for severity grading of artery hardening. Our method can find and validate possible arterio-venous crossing points, for which the severity grade is predicted. (ii) We design a new model, MDTNet, which uses the focal loss to address the problem of data ambiguity and unbalance.

## Dataset

### Ethics statement

This study was performed in accordance with the World Medical Association Declaration of Helsinki. Patients gave written informed consent to participate and the study protocol was approved by the institutional review board of the Osaka University Hospital.

We built a vessel crossing point dataset extracted from our retinal image database of the Ohasama study, a cohort to study cardiovascular diseases risk, where we could utilize 1, 440 images in the size of 5, 184 × 3, 456 pixels, which are captured by the CR-2 AF Digital Non-Mydriatic Retinal Camera (Canon, Tokyo) between 2013 and 2017 as JPEG files. This database includes the medical data of 684 people, which are with an average age of 64.5 (standard deviation: 6.1). The ratio between female and male is 65.2% : 34.8% and 47.6% of all participants have hypertension. Details of the study profile were published elsewhere [16].

**Fig 2:**
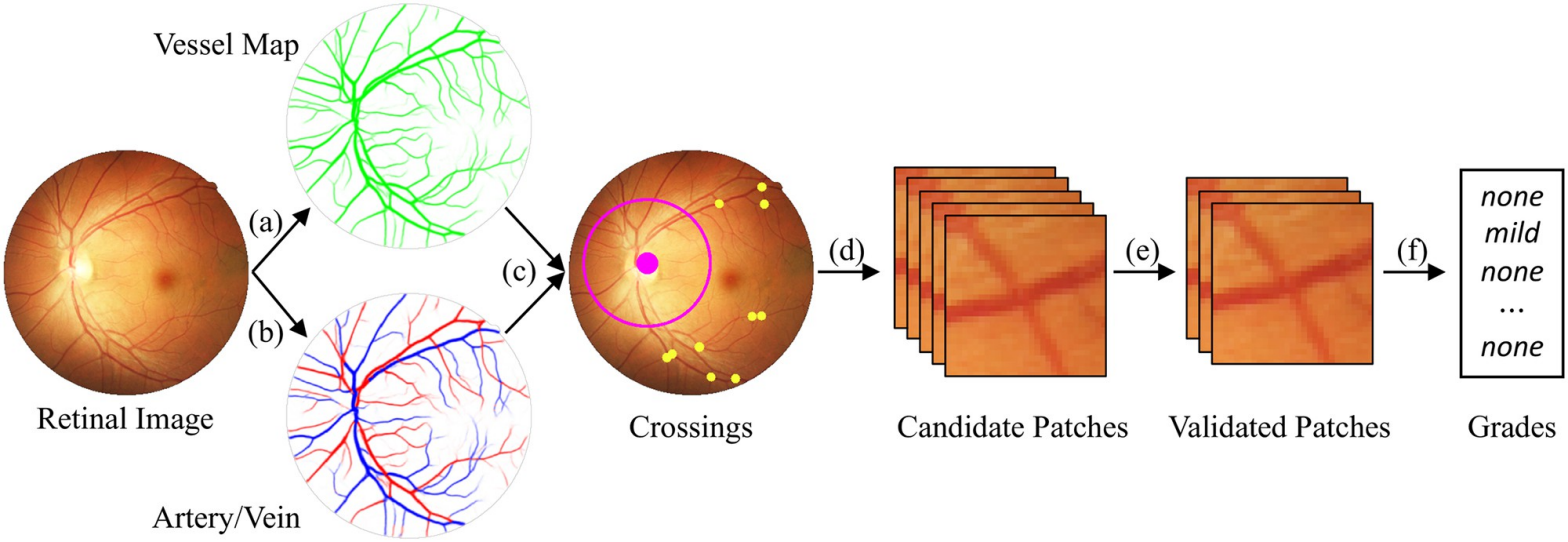
Overall pipeline of our severity grading.

To find crossing points in these images (Fig 2(a)–2(d)), we used a segmentation model ([12]) to get vessel maps. We then classified each pixel on extracted vessels into artery/vein using [17]. We combine the vessel segmentation and classification results to find crossing points because classification results, which are more beneficial for crossing point detection, tend to have more errors while segmented vessel maps are more accurate. Therefore, we refine the classification results based on the vessel maps. A classic approach then finds crossing points in these refined artery/vein maps. Specifically, we find the artery pixels neighbouring vein pixels and check whether it is a crossing point or not using the skeletonized vessel map. The points marked in yellow in Fig 2 are detected crossing point candidates. Note that for cup zones as indicated by a pink circle and dot in Fig 2, we exclude candidates because the vessel system in this area is with high complexity and thus segmentation and classification are not reliable. Image patches are of size 150 × 150, centered at the crossing point candidates. Consequently, we detected 4, 240 crossing points and extracted corresponding image patches, centered at these crossing points.

Each image patch was carefully reviewed by a highly experienced ophthalmologist. Due to the errors in vessel segmentation and artery/vein classification, the detected crossing points may not be actual or informative. Therefore, the specialist first annotated each image patch with a label on its validity, *i.e*., if the image patch contains an actual and informative crossing point (*true*) or not (*false*). The numbers of true and false crossing points are 2, 507 and 1, 733, respectively. For each true crossing point, the specialist gave its severity label in *C* = {*none*, *mild*, *moderate*, *severe*}. The numbers of image patches with respective labels are 1, 177, 816, 457, and 57. In both tasks, the datasets will be divided into training, validation, and test sets following a ratio of 8:1:1. As an examinee may have multiple retinal images, it is important to strictly put them into one same subset to prevent the training data contamination.

## Severity grading pipeline

Our method forms a pipeline with three main modules, *i.e*., preprocessing, patch validation, and severity grade prediction. The whole pipeline is shown in Fig 2.

### Preprocessing

Steps (a)–(d) in the figure are preprocessing, in which the same processes as our dataset construction are applied to get image patches of 150 × 150 pixels with crossing point candidates.

### Crossing point validation

Both crossing point validation and severity grading are classification problems, whereas validation is easier because the label distribution is more balanced and the differences between real and false crossing points are more obvious. We find that commonly used classification models, such as [18–20], work well for our validation task (refer to *Experiments and Results* Section).

### Severity grade prediction

The severity grade prediction task is much more challenging: Firstly, the label distribution is highly biased. For example, samples with the *none* label account for 68% of the total samples, while ones with the *severe* label only take up 3%. Secondly, the difference among samples with different labels may not be clear enough. Even medical doctors may make diverse decisions on a single image patch.

For such classification tasks with ambiguous or imbalanced classes, the focal loss [15] has been used, which makes a model more aware of hard samples than easy ones. The focal loss introduces a hyperparameter *γ*, on which a model’s performance depends significantly. Tuning this hyperparameter is extremely important yet computationally expensive [21]. A greater *γ* may make the model focus too much on hard samples, spoiling the accuracy of other samples, while a smaller *γ* may decrease its ability to classify hard samples.

**Fig 3:**
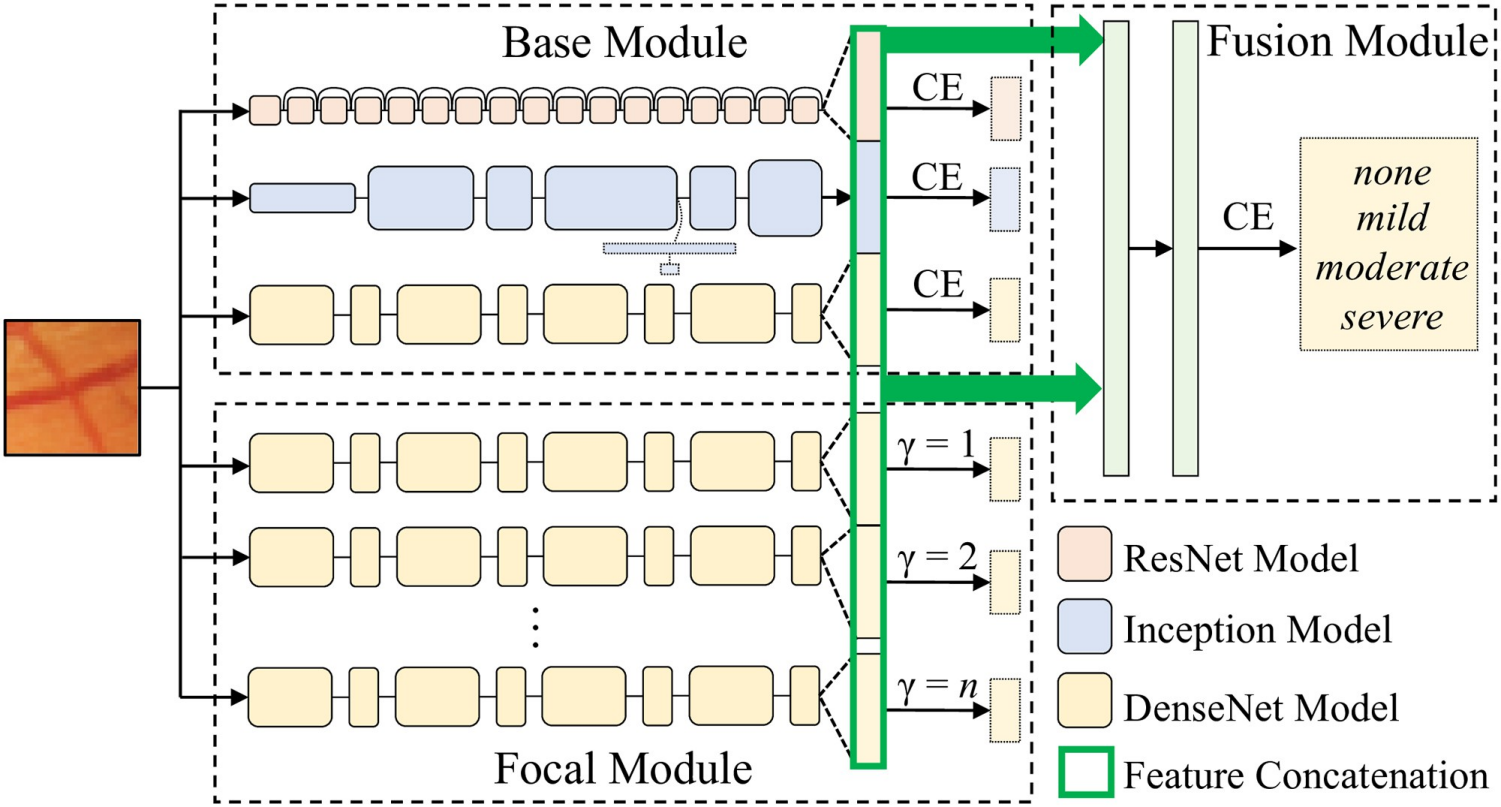
MDTNet for severity grade prediction.

We propose a multi-diagnosis team network (MDTNet) to address the aforementioned problems in severity grade prediction. As shown in Fig 3, MDTNet consists of three modules, *i.e*., a base module, a focal module, and a fusion module.

The base and focal modules have multiple sub-models, and all of them take the same image patch as input. The difference between the sub-models in the base and focal modules is the losses: Ones in the base module adopt the cross entropy (CE) loss while ones in the focal module use the focal loss. These sub-models are trained independently with respective losses. The fusion module concatenates all features (*i.e*., the outputs of the second last layers of the sub-models) into a single vector, which is then fed into two fully-connected layers to make the final prediction.

The focal loss is originally designed for object detection [15], defined as

$$\begin{array}{c} L\left(y,t\right)=-\sum_{l}t_{l}{\left(1-y_{l}\right)}^{\gamma }\;\text{log}\;y_{l}, \end{array}$$ (1)

where *t* is the one-hot representation of label and *y* is the softmax output from a model (*t*_*l*_ and *y*_*l*_ are the *l*-th entries of *t* and *y*); *γ* is a hyperparameter to weight hard examples. The focal loss reduces to the CE loss when *γ* = 0, and a larger *γ* weights more on hard examples. One possible criticism of the focal loss is its sensitivity to *γ*. We therefore propose to ensemble sub-models with different *γ*’s. The hypothesis behind this choice is that different *γ*’s may rely on different cues for prediction and aggregating respective features may help in improving the final decision. This is embodied in the focal module. The same idea can also be applied to different network architectures, embodied in the base module. These sub-models thus provide diagnostic features that may complement each other.

To cope with the imbalanced class distribution, we adopt class weighting [22, 23]. We multiply weight *α*_*l*_ = ln *N*_*l*_/ln *N* to each term (*i.e*. different *l*’s) in the CE/focal loss, where *N* and *N*_*l*_ are the numbers of all samples and of samples with the label corresponding to the *l*-th entry of *t*. We pre-train the sub-models using their own classifiers and losses, and then freeze their weights to train the additional two fully-connected layers for the final decision.

### Data augmentation

**Fig 4:**
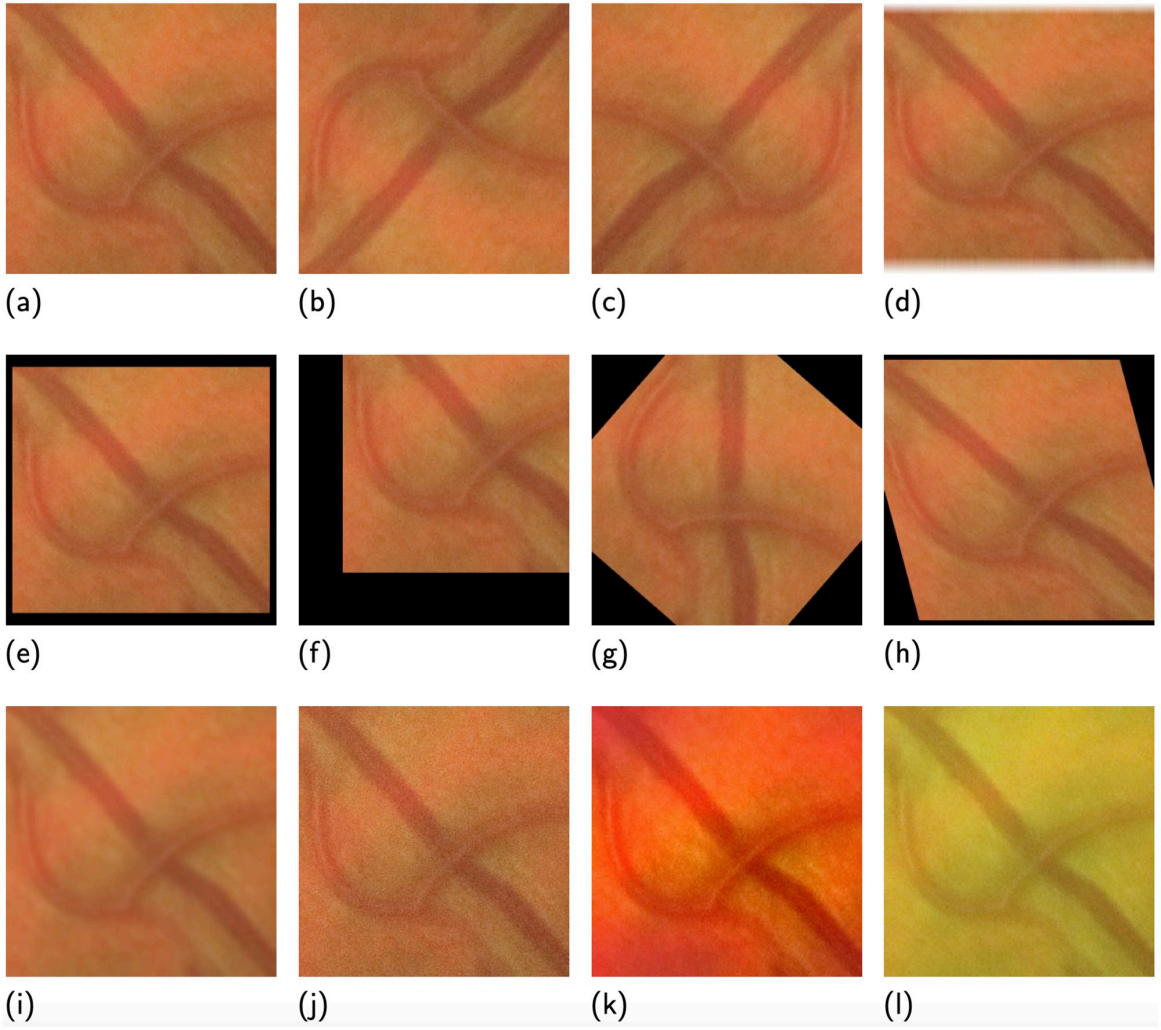
Our data augmentation operator pool. (a) Raw image, (b) vertical flipping, (c) horizontal flipping, (d) cropping and padding, (e) scaling, (f) translating, (g) rotating, (h) sheering, (i) blurring, (j) additional noise, (k) additional frequency noise, and (l) color modification.

We adopt extensive data augmentation. During the training process, the input images have 50% chance of getting each operator in Fig 4. Among them, (b∼h) are used for shape modification, changing the locations and the shapes of the attention areas of the deep learning models; (i∼k) are to provide variety on imaging quality by blurring or adding random noises; (l) represents sensor characteristics of color (hue and saturation).

## Experiments and results

### Implementation

For sub-models in the base module, we used ResNet [18], Inception [20], and DenseNet [19]. In the focal module, DenseNet with *γ* = 1, 2, or 3 were used. All these models are pre-trained over the ImageNet dataset [24]. The fully-connected layers in the fusion module are followed by the ReLU nonlinearity. For optimization, Adam [25] was adopted with a learning rate of 0.0001. Models are trained on the training set, and the weights with the highest performance on the validation set are selected as the best models, which will be evaluated on the test set.

### Performance of base models

We first evaluated the performance of the base module’s sub-models for the crossing point validation and severity grade prediction tasks. For comparison, we also give the results of models without pre-training (w/o PT) and without data augmentation (w/o DA), as well as models using only the green channel (GC Only).

The crossing point validation performances are shown in the left part of Table 1. We use two metrics, precision and recall, and the time measurement to show the timing performance. We can see that pre-training and data augmentation can improve the overall performance of the crossing point validation. The Inception model with PT and DA achieved the best recall and the second-best precision. Note that PT and DA will not change the running time of the model because they do not modify the network structure.

**Table 1:** Performances of base models with ablation.

Models	Cross. Point Val.			Severity Grade Pred.		
	Pre.	Rec.	*t* (ms)	Acc.	Kappa	*t* (ms)
ResNet-50	0.9427	0.9526	0.274	0.8063	0.6629	0.278
—w/o PT	0.8646	0.6975	0.274	0.5445	0.0177	0.278
—w/o DA	0.9531	0.8551	0.274	0.5340	0.0036	0.278
—GC Only	0.9583	0.9154	0.273	0.7277	0.5288	0.273
Inception v3	0.9635	**0.9635**	0.218	0.8534	0.7432	0.222
—w/o PT	0.9010	0.6865	0.218	0.5183	0.0313	0.222
—w/o DA	0.9323	0.9179	0.218	0.5393	0.0000	0.222
—GC Only	0.9167	0.9119	**0.216**	0.8115	0.6771	**0.216**
DenseNet-121	0.9479	0.9630	0.266	**0.8795**	**0.7892**	0.269
—w/o PT	0.9375	0.6742	0.266	0.5288	0.0050	0.269
—w/o DA	**0.9740**	0.8274	0.266	0.7225	0.4865	0.269
—GC Only	**0.9740**	0.9212	0.266	0.6702	0.4406	0.267

The right part of Table 1 gives the results of the base models on the severity grade prediction task, and Table 2 presents the performance of MDTNet and models using the focal loss. In addition to the classification accuracy, we also adopt Cohen’s kappa, which can measure the agreement between the ground-truth labels and predictions. We can see that, compared with the focal loss models, the DenseNet can achieve higher overall accuracy with the CE loss. However, the combination of different models, different losses, as well as different *γ* values can boost the performance. MDTNet achieved the highest performance in this experiment when *n* = 3.

**Table 2:** Performance of MDTNet models for severity grade prediction.

Metrics	DenseNet-121 (Focal Loss)				MDTNet		
	*γ* = 1	*γ* = 2	*γ* = 5	*γ* = 10	*n* = 0	*n* = 1	*n* = 3
Acc.	0.8639	0.7434	0.8639	0.7958	0.8953	0.9110	**0.9162**
Kappa.	0.7642	0.5685	0.7641	0.6508	0.8183	0.8453	**0.8542**
*t* (ms)	**0.268**	**0.268**	**0.268**	**0.268**	0.767	1.047	1.571

**Fig 5:**
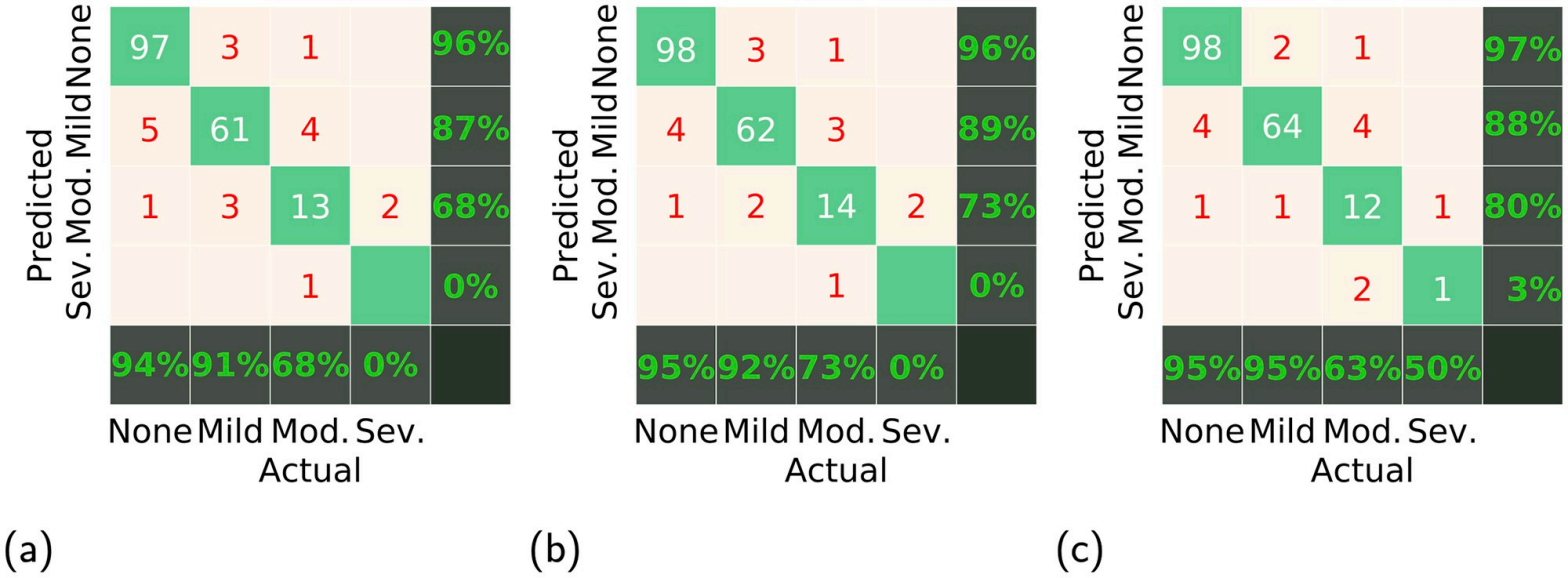
Confusion matrices for three different severity grade prediction models. The recall is shown in the last row and the precision is shown in the last column. (a) MDTNet without the focal module, (b) MDTNet for *n* = 1, and (c) MDTNet for *n* = 3.

**Fig 6:**
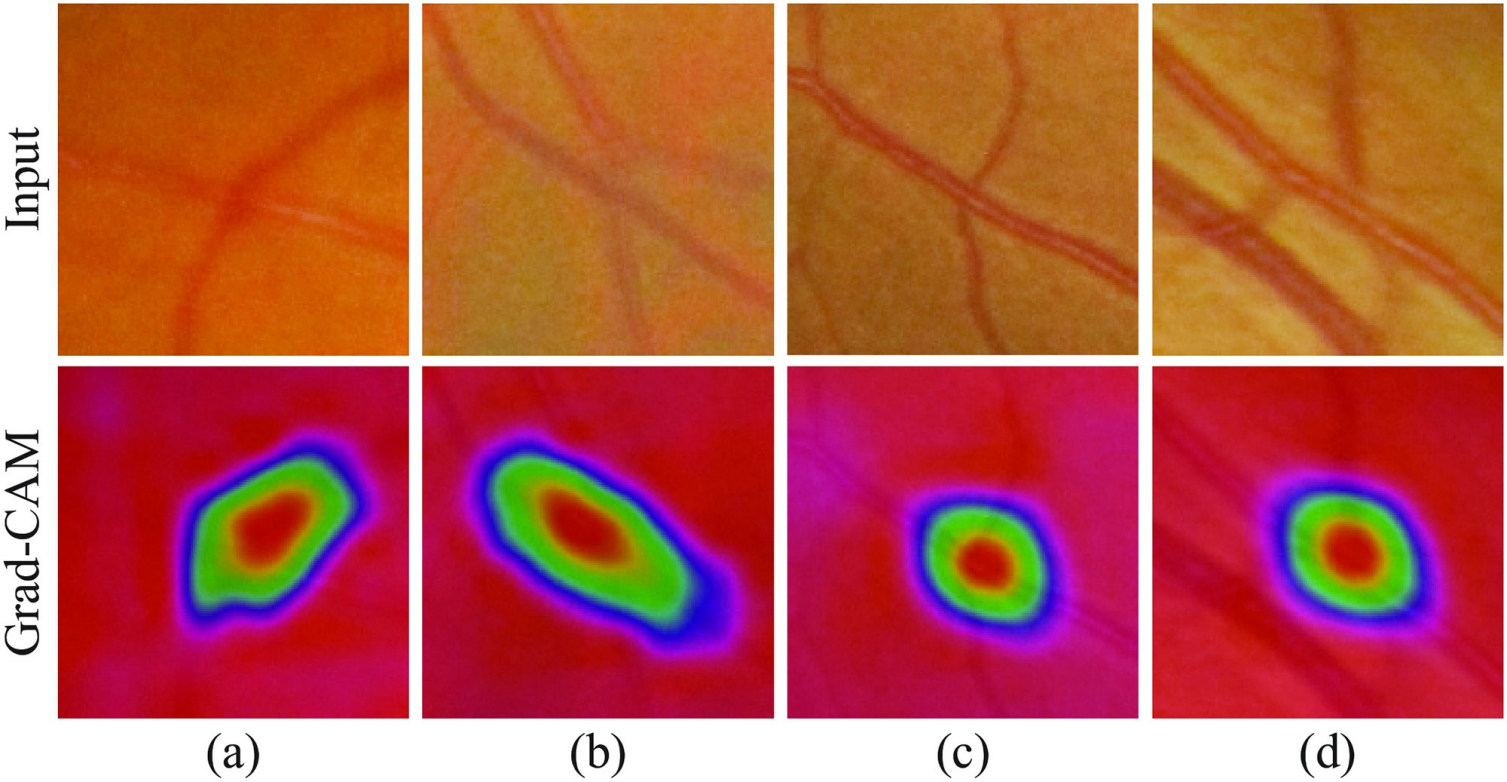
Visual explanation of prediction results. (a,b) are for the crossing point validation model and (c,d) are from the severity grade prediction model. The first row is the raw input images and the second row is the class-discriminative regions.

To better analyze the severity grade prediction performance, we present the confusion matrices in Fig 5. It can be seen that, with the increment of the underlying sub-models, MDTNet gains the classification ability. Fig 6 shows visual explanation of MDTNet by Grad-CAM [26]. Fig 6 (a) and 6(b) show two examples for the crossing point validation. The ground-truth labels are *false* and the predictions were also *false*, *i.e*., these are not effective crossing points as the arteries are under the veins. The model mainly counted the red area in the second row along the vein. The model might find the vein, track it down, and reach the conclusion that it lies above the artery. Fig 6 (c) and 6(d) are for the severity grade prediction. The ground-truth labels are respectively *mild* and *moderate* and were both correctly predicted. We can see the artery runs over the vein deforming the vein. Being different from the example in (a) and (b), the model looks at the crossing points and looks for possible shape deformations and their extent.

## Conclusion

The paper presents a method to automatically classify the arteriolosclerosis severity from retinal images following ophthalmologists’ diagnostic process. To improve the accuracy for ambiguous and unbalanced samples, we design the multi-diagnosis team network (MDTNet), which can jointly consider diagnostic cues from multiple sub-models, without tuning the hyperparameter for the focal loss. Experimental results show the superiority of our method, achieving over 91% accuracy. Most importantly, the whole process can be checked to see how the grading was determined as it is designed to be a step-by-step approach replicating ophthalmologists’ diagnostic process. Therefore, the proposed method can serve as a supporting tool for experienced ophthalmologists to efficiently grade the images in a consistently reproducible manner. A quality checklist [27] for the proposed deep learning method is shown in Table 3.

**Table 3:** The MI-CLAIM checklist.

Before paper submission		
Study design (Part 1)	Page	Notes
The clinical problem in which the model will be employed is clearly detailed in the paper.	2-3	
The research question is clearly stated.	3	
The characteristics of the cohorts (training and test sets) are detailed in the text.	3-4	
The cohorts (training and test sets) are shown to be representative of real-world clinical settings.	3-4	
The state-of-the-art solution used as a baseline for comparison has been identified and detailed.	7-8	
Data and optimization	Page	Notes
The origin of the data is described and the original format is detailed in the paper.	3-4	
Transformations of the data before it is applied to the proposed model are described.	6-7	
The independence between training and test sets has been proven in the paper.	4	
Details on the models that were evaluated and the code developed to select the best model are provided.	7	
Is the input data type structured or unstructured?	☑ Structured	□ Unstructured
Model performance (Part 4)	Page	Notes
The primary metric selected to evaluate algorithm performance, including the justification for selection, has been clearly stated.	7-8	
The primary metric selected to evaluate the clinical utility of the model, including the justification for selection, has been clearly stated.	7-8	
The performance comparison between baseline and proposed model is presented with the appropriate statistical significance.	7-9	
Model examination (Part 5)	Page	Notes
Examination technique	7-9	
A discussion of the relevance of the examination results with respect to model/algorithm performance is presented.	7-9	
A discussion of the feasibility and significance of model interpretability at the case level if examination methods are uninterpretable is presented.	8	
A discussion of the reliability and robustness of the model as the underlying data distribution shifts is included.	4,7-9	
Reproducibility (Part 6): choose appropriate tier of transparency		Notes
Tier 1: complete sharing of the code	☑	
Tier 2: allow a third party to evaluate the code for accuracy/fairness; share the results of this evaluation	□	
Tier 3: release of a virtual machine (binary) for running the code on new data without sharing its details	□	
Tier 4: no sharing	□	
